# Post-traumatic stress disorders among children and adolescents in conflict-affected zones of Amhara region, February 2022

**DOI:** 10.3389/fpsyg.2022.1052975

**Published:** 2023-01-04

**Authors:** Gebeyaw Biset, Debrnesh Goshiye, Negesse Melesse, Mekonnen Tsehay

**Affiliations:** ^1^Department of Pediatrics and Child Health Nursing, School of Nursing and Midwifery, College of Medicine and Health Sciences, Wollo University, Dessie, Ethiopia; ^2^Dream Science and Technology College, Dessie, Ethiopia; ^3^Department of Psychiatric Nursing, School of Nursing and Midwifery, College of Medicine and Health Sciences, Wollo University, Dessie, Ethiopia

**Keywords:** conflict, displacement, post-traumatic stress disorder, Ethiopia, Amhara region

## Abstract

**Background:**

To date, conflict is causing extreme social crises worldwide, with children representing the most vulnerable group, often experiencing severe trauma and violence in conflict-ridden areas. Posttraumatic stress disorders (PTSDs) are the most widely reported psychological problem in the aftermath of conflict. However, less attention has been given to children living in conflict-prone areas of the world.

**Objective:**

The present study aimed to assess posttraumatic stress disorders among children and adolescents in conflict-affected zones of the Amhara region in Ethiopia from January to February 2022.

**Method:**

A community-based cross-sectional study was employed from January to February 2022. A multistage random sampling technique was employed to recruit the study participants. A structured interviewer-administered questionnaire was designed to collect the desired data. Data were verified, coded, and entered into EpiData version 3.1 and analyzed using SPSS version 24 statistical software.

**Result:**

A total of 846 children with a response rate of 94.33% were screened for trauma. Subsequently, 557 (69.80%) children had experienced trauma. Of these 557 children who had experienced trauma, 203 (36.45%) developed posttraumatic stress disorders. Based on these results, this study recommends that mass screening of children and adolescents for posttraumatic stress disorders and rehabilitation services be undertaken, including resilience training and psychosocial support.

## Introduction

Conflict is rising to an alarming degree worldwide, affecting the lives of millions of civilians, particularly children and adolescents (Leatherman, 2011; Rieder and Choonara, 2012; Østby et al., 2020; Bendavid et al., 2021). According to a report by the United Nations International Children's Emergency Fund (UNICEF), more than 230 million children are living in conflict-ridden regions all over the world. More than 170,000 grave human rights violations against children have been detected and verified since 2010, representing more than 45 human rights violations per day against children (Kadir et al., 2018). Children in conflict-ridden zones are at increased risk of death, injuries, disability, illness, psychological trauma, and social and cultural disintegration (Santa Barbara, 2006). Additionally, children experience the horror of watching their parents or other members of society fight, flee, or die in conflict, which deeply affects their attitude toward society and their future social relationships (Macksoud, 1994).

Conflict-induced population displacement is becoming the greatest human tragedy in the world. In 2019, globally, as many as 45.7 million people were displaced from their homeland due to conflict and human rights violations (United Nations High Commissioner for Refugees (UNHCR), 2020; Draper, 2021). The majority of the internally displaced people are located in Africa and who are severely attacked by the conflict (United Nation High Commissioner for Refuegges (UNHCR's), 2020–2021). Population displacement is the most significant contemporary problem facing Ethiopia today. A significant portion of this displacement is conflict induced, which is largely related to ethnic and border-based disputes (Internal Displacment Monitoring Center (IDMC), 2019; International Organization for Migration Displacement Tracking Matrix (IOM-DTM), 2019; Yigzaw and Abitew, 2019). Studies have revealed that displaced children are at a higher risk of violence and facing mental health problems (Usta et al., 2008; Reed et al., 2012; Al-Natour et al., 2019).

Posttraumatic stress disorder (PTSD) is one of the most widely reported psychological problems experienced by a person in the aftermath of any gruesome conflict (Van der Kolk et al., 1996; Catani, 2018). PTSD is characterized by symptoms of intrusion, avoidance, changes in mood or cognition, and hyperarousal, all of which lead to considerable social and interpersonal dysfunction in the affected individual (Roberts and Browne, 2011; Sareen, 2018). Posttraumatic stress disorders are experienced as a consequence of witnessing or being directly involved in terrible events such as murder, threats, or loss of family or belongings (Ohayon and Shapiro, 2000; Thabet and Vostanis, 2000; Attanayake et al., 2009; Murad, 2019). The majority of PTSD cases are reported in developing countries (Steel et al., 2009; Morina et al., 2018; Bapolisi et al., 2020; Madoro et al., 2020; Ng et al., 2020); however, treatment-seeking behavior is low in developing countries compared to high-income countries (Koenen et al., 2017).

The likelihood of posttraumatic stress disorder and the presentation of PTSD symptoms are affected by a number of individual and societal factors. Social disadvantage, being female, unmarried, having a younger age, personal and family psychiatric history, lower educational level, and lower socioeconomic status are all factors associated with a higher risk of PTSD among trauma-exposed children (Kroll, 2003; Stein et al., 2007; Sareen et al., 2013; Siriwardhana et al., 2013; Benjet et al., 2016; Mahmood et al., 2019; Malejko et al., 2020).

Posttraumatic stress disorders (PTSDs) have deleterious consequences on the health of children and adolescents. Moreover, children with PTSD can suffer from depression and anxiety, substance abuse, antisocial behaviors, stunted brain development, and poor school performance (Benjet et al., 2016; Malejko et al., 2020). The findings of the present study provide insights into the extent of mental destruction that PTSD can cause in children and adolescents in the conflict zones of the Amhara region, which, in turn, will help to take interventional measures.

## Materials and methods

### Study setting and study populations

The present study is a community-based cross-sectional study conducted in the conflict-affected zones of the Amhara region from January to February 2022. The inclusion criteria for the study were as follows: (1) children aged under 18 years old and (2) children who had lived in conflict zones in the Amhara region. The exclusion criteria for the present study were as follows (1) children who had already left conflict-ridden zones in the Amhara region before the commencement of conflict for other reasons and returned only after the conflict ended and (2) children with hearing impairment or severe illness. A multistage random sampling method was used to select the study participants.

### Sample size determination

The sample size was determined using the single population proportion formula by considering the assumptions *Z*α/2 = critical value for normal distribution at 95% confidence level (CI) that equals 1.96 (*z* value at α = 0.05), estimated proportion (*p*) of 50%, and absolute precision or margin of error 5% (*d* = 0.05). The following formula was used to calculate the sample size:


$$\begin{array}{l} no=\frac{{\left(\frac{Za}{2}\right)}^{2}\;*\;p\left(1-p\right)}{d^{2}}\\ no=\frac{{\left(1.96\right)}^{2}\;*\;0.5\left(1-0.5\right)}{0.05^{2}}\\ no=384. \end{array}$$

Considering 10% of the non-respondents and multiplying by the design effect of 2, the final sample included 846 participants.

### Data collection tool and procedure

The child PTSD symptoms scale self-report for DSM V (Diagnostic and Statistical Manual of Mental Disorders, Fifth Edition; CPSS-SR) was used to assess PTSD among children and adolescents (Weathers et al., 2013; Foa et al., 2018). The 20 child PTSD symptoms scale self-report (CPSS-SR) items were rated on a 5-point scale of frequency from 0 (not at all) to 4 (6 or more times a week/severity). The sum of all yes responses for 20-item questions greater than 31 was used as the cutoff point for the PTSD assessment. In addition, the severity scale of PTSD was classified as: “0–10” minimal, “11–20” mild, “21–40” moderate, “41–60” severe, and “61–80” very severe.

Data were collected through face-to-face interviews with adolescents aged 8 years and over, and child guardians whose children were aged below 8 years old using the 20 self-report items. A total of four data collectors and two supervisors were involved during the entire data collection period. Prior to the data collection period, the data collectors and supervisors were trained for two days. The completeness of data was checked daily during the entire data collection period. A pretest was undertaken on 5% of the sample size before the actual data collection time.

### Data processing and analysis

Data were verified, transformed, coded, entered into EpiData version 3.1, and analyzed using SPSS version 24 Software. The descriptive statistics were computed and presented using frequencies, proportion, and tables.

## Results

### Sociodemographic characteristics of the participants

This study included 846 children and adolescent participants with a response rate of 94.33%. In total, 408 (51.13%) children were aged <8 years and 390 (48.87%) were aged 8–17 years. The mean age of the children as participants was 8.45 years with a standard deviation (SD) of 5 years. Nearly one-third of the children (64.42%) were urban residents (Table 1).

**Table 1:** Sociodemographic feature of children and adolescents in the conflict-affected region of Amhara region June 2022.

**Variable**	**Category**	**Frequency**	**Percentage**
Age of the child	<5 years	325	40.73
	6–11 years	148	18.55
	11–17 years	325	40.73
Sex of the child	Male	460	57.64
	Female	338	42.36
Residence of the child	Rural	286	35.84
	Urban	512	64.42

### Trauma among children

Of all the participants, 557 (69.80%) children and adolescents had experienced trauma during conflict. In total, 423 (53.01%) children had experienced physical trauma, 535 (67.04%) psychological trauma, and 96 (12.03%) had experienced sexual trauma/violence.

### PTSD among children and adolescents

Children and adolescents who had experienced trauma during the conflict were eligible to be assessed for PTSD. Thus, 557 children were assessed for posttraumatic stress symptoms. As many as 203 (36.45%) children had developed posttraumatic stress disorders following the trauma (Table 2). Of these, 77 (13.82%) had minimal PTSD, 125 (22.44%) had mild PTSD, 246 (44.17%) had moderate PTSD, 85 (15.26%) had severe PTSD, and 24 (4.31%) had very severe PTSD symptoms.

**Table 2:** Posttraumatic stress disorder among children and adolescents in the conflict-affected region of Amhara region June 2022.

**Post-traumatic stress disorders (PTSD) among children and adolescents**	**Not at all**	**Once a week**	**2 or 3 times a week**	**4 or 5 times a week**	**More than 6 times a week**
	***n*** **(%)**	***n*** **(%)**	***n*** **(%)**	***n*** **(%)**	***n*** **(%)**
Intrusive/upsetting thoughts or pictures	158 (28.37)	122 (21.90)	100 (17.95)	114 (20.47)	63 (11.31)
Having bad dreams or nightmare	159 (28.55)	88 (15.80)	122 (21.90)	159 (28.01)	32 (5.75)
Acting/feeling as if it was happening again	247 (44.34)	99 (17.77)	69 (12.39)	105 (18.85)	37 (6.64)
Feeling upset when remembering the event	140 (25.13)	76 (13.64)	114 (20.47)	170 (30.51)	57 (10.23)
Body feelings when remembering the event	311 (55.83)	87 (15.62)	94 (16.88)	29 (5.21)	36 (6.46)
Trying not to think about the event	197 (35.37)	44 (7.90)	41 (7.36)	56 (10.05)	219 (39.32)
Stay away from the reminders of the event	247 (44.34)	57 (10.23)	54 (9.69)	42 (7.54)	157 (28.19)
Not able to remember an important part of event	189 (33.93)	51 (9.16)	41 (7.36)	25 (4.49)	251 (45.06)
Having bad thoughts for self and others	340 (61.06)	42 (7.54)	64 (11.49)	32 (5.75)	79 (14.18)
Thinking what happened is self-fault	354 (63.55)	63 (11.31)	64 (11.49)	39 (7.00)	37 (6.64)
Having a strong bad feeling	246 (44.17)	90 (16.16)	97 (17.41)	104 (18.67)	20 (3.59)
Loss of interest in doing things	316 (56.73)	99 (17.77)	57 (10.23)	58 (10.41)	27 (4.85)
Not feeling close to family and friends	343 (61.58)	78 (14.00)	54 (9.69)	55 (9.87)	27 (4.85)
Trouble of having good feelings	332 (59.61)	88 (15.80)	61 (10.95)	43 (7.72)	33 (5.92)
Getting angry easily	309 (55.48)	84 (15.08)	76 (13.64)	50 (8.98)	38 (6.82)
Doing things that might hurt self	363 (65.17)	51 (9.16)	44 (7.90)	51 (9.16)	48 (8.62)
Being very careful/on the lookout for danger	218 (39.14)	69 (12.39)	57 (10.23)	93 (16.70)	120 (21.54)
Being jumpy or easily scared	161 (28.90)	128 (22.98)	100 (17.95)	100 (17.95)	68 (12.21)
Having trouble of paying attention	270 (48.47)	135 (24.24)	66 (11.85)	50 (8.98)	36 (4.46)
Having trouble falling or staying sleep	199 (35.73)	137 (24.60)	103 (18.49)	84 (15.08)	34 (6.10)

## Discussion

Studies have shown that 4% of children under the age of 18 are exposed to some form of traumatic event during their lifetime that leads to posttraumatic stress disorders. Children and adolescents living in areas affected by conflict, displaced children, and children in refuges are at a higher risk of trauma and violence, which can lead to them suffering from posttraumatic stress disorders. Posttraumatic stress disorders have a detrimental effect on children and adolescents living in conflict zones. In addition, children with PTSD are at a higher risk of experiencing other mental health problems that include depression, anxiety, suicidal thought, stunted brain development, and poor school performance (AlShawi, 2018).

More than one-third (36.45%) of children living in conflict-ridden areas have experienced posttraumatic stress disorders. The findings of the present study are similar to that of others conducted in developing countries, which showed that half of trauma-exposed children developed posttraumatic stress disorders (Kessler et al., 1995; Fariba and Gupta, 2020). Similarly, the results of the present study are comparable to findings from India, where 30.6% of children experienced posttraumatic stress disorders (Kar et al., 2007). The high prevalence of PTSD might be attributed to the fact that children and adolescents have less emotional and intellectual capability to cope with traumatic events and are more likely to experience posttraumatic stress disorders compared to other groups in society.

The findings of the present study are higher than those of a study undertaken among internally displaced persons in Iraq (20.8%) (AlShawi, 2018). They also differ from those of a study undertaken among children aged under 18 years old (10%) in developing countries (Fariba and Gupta, 2020), and that of another study undertaken among children in Greece (16%) (Kolaitis, 2017). A possible explanation for this discrepancy could be attributed to the fact that the children exposed to different levels of traumatic events might experience different levels of posttraumatic stress disorders. In addition, the discrepancy could be associated with the different levels of psychosocial support provided to children and adolescents living in areas affected by conflict in different countries.

On the other hand, the findings of the present study were lower than those of a study among refugees in Nakivale camp in southwestern Uganda (67%) (Bapolisi et al., 2020). They were also lower than those of a study among Syrian refugees residing in the Kurdistan region of Iraq (60%) (Mahmood et al., 2019), and a study on displaced populations in Ethiopia (58.4%) (Madoro et al., 2020). This difference could be attributed to the variations in the intensity of the traumatic events experienced by the study populations. In addition, the differences between these findings could be due to the different data collection procedures used in these studies. In the current study, child legal guardians were the source of information for symptoms of PTSD in the children, which could lower the prevalence of actual PTSD.

## Conclusion and recommendations

The findings of the present study showed that more than one-third of children and adolescents had experienced posttraumatic disorders due to witnessing, facing, or experiencing traumatic events during conflict. Of these, one in eight children had a severe form of posttraumatic stress disorder. The government and international humanitarian aid agencies should provide care and attention to children in conflict zones. Mass screening, psychosocial support, and resilience training programs are recommended for children and adolescents in all war-affected zones.

## Data availability statement

The original contributions presented in the study are included in the article/supplementary material, further inquiries can be directed to the corresponding author.

## Ethics statement

The studies involving human participants were reviewed and approved by Dream Science and Technology College. Written informed consent to participate in this study was provided by the participants' legal guardian/next of kin.

## Author contributions

GB, DG, NM, and MT conceived and designed the study, read, and approved the manuscript. DG and NM performed data cleaning, editing, entry, and wrote the first draft of the manuscript. GB and MT conducted data analyses and wrote the result. GB was responsible for the submission of the manuscript. All authors contributed to the article and approved the submitted version.
